# Toxicokinetics, including saturable protein binding, of 4-chloro-2-methyl phenoxyacetic acid (MCPA) in patients with acute poisoning

**DOI:** 10.1016/j.toxlet.2011.01.011

**Published:** 2011-03-25

**Authors:** Darren M. Roberts, Andrew H. Dawson, Lalith Senarathna, Fahim Mohamed, Ron Cheng, Geoffrey Eaglesham, Nick A. Buckley

**Affiliations:** a South Asian Clinical Toxicology Research Collaboration, University of Peradeniya, Peradeniya, Sri Lanka^1^; b Burns, Trauma and Critical Care Research Centre, Royal Brisbane and Women's Hospital, University of Queensland, Brisbane, Queensland, Australia; c Professorial Medicine Unit, POWH Clinical School, University of New South Wales, Randwick, New South Wales, Australia; d New South Wales Poisons Information Centre, The Children's Hospital, Westmead, New South Wales, Australia; e Organic Chemistry, Queensland Health Forensic and Scientific Services, Brisbane, Queensland, Australia

**Keywords:** MCPA, 4-chloro-2-methylphenoxyacetic acid, 2,4-D, 2,4-dichlorophenoxyacetic acid, *t*_1/2_, apparent elimination half-life, Cu, free (unbound) plasma concentration, Kd_*i*_, affinity constant of binding at the *i*th site, Bmax_*i*_, maximum density (concentration of saturation) of binding at the *i*th site, *C*_i_, initial concentration, *C*_*t*_, concentration after time *t*, *k*, elimination rate constant, LOD, limit of detection, LOR, limit of reporting, Tmax, time of the maximum plasma concentration, IQR, interquartile range, Koc, octanol solubility coefficient, p*K*_a_, acid dissociation constant, CL, clearance, Vd, volume of distribution, Chlorophenoxy herbicide, 4-Chloro-2-methylphenoxyacetic acid (MCPA), Toxicokinetic, Protein binding, Dose-dependent

## Abstract

Human data on protein binding and dose-dependent changes in toxicokinetics for MCPA are very limited. 128 blood samples were obtained in 49 patients with acute MCPA poisoning and total and unbound concentrations of MCPA were determined. The Scatchard plot was biphasic suggesting protein binding to two sites. The free MCPA concentration increased when the total concentration exceeded 239 mg/L (95% confidence interval 198–274 mg/L). Nonlinear regression using a two-site binding hyperbola model estimated saturation of the high affinity binding site at 115 mg/L (95%CI 0–304). Further analyses using global fitting of serial data and adjusting for the concentration of albumin predicted similar concentrations for saturable binding (184 mg/L and 167 mg/L, respectively) without narrowing the 95%CI. In 25 patients, the plasma concentration–time curves for both bound and unbound MCPA were approximately log-linear which may suggest first order elimination, although sampling was infrequent so zero order elimination cannot be excluded. Using a cut-off concentration of 200 mg/L, the half-life of MCPA at higher concentrations was 25.5 h (95%CI 15.0–83.0 h; *n* = 16 patients) compared to 16.8 h (95%CI 13.6–22.2 h; *n* = 10 patients) at lower concentrations. MCPA is subject to saturable protein binding but the influence on half-life appears marginal.

## 1 Introduction

The chlorophenoxy compounds 4-chloro-2-methylphenoxyacetic acid (MCPA) and 2,4-dichlorophenoxyacetic acid (2,4-D) are selective herbicides used in agricultural and household sectors worldwide. 2,4-D is the most commonly used chlorophenoxy herbicide in the US (Kiely et al., 2004) and acute self-poisoning with MCPA is a common reason for presentation to rural hospitals in Sri Lanka where subsistence farming is common (Roberts et al., 2005). Severe poisoning including coma, rhabdomyolysis and renal toxicity may occur and persist for some days. Death occurs in around 5% of patients and is typically 24–48 h post-ingestion (Roberts et al., 2005). The mechanism of fatal toxicity has not been defined (Roberts et al., 2005). Animal studies have also suggested that prolonged elimination of chlorophenoxy herbicides leads to increased toxicity (Timchalk, 2004). Further, saturation of protein binding increases the free (unbound) concentration of the poison, which is then available to distribute from the plasma (central) compartment. In the case of chlorophenoxy compounds, this is important because the mechanism of toxicity is thought to relate to disruption of intracellular processes (Roberts and Buckley, 2007a).

The treatment of acute chlorophenoxy herbicide poisoning consists of decontamination of the gastrointestinal tract, resuscitation and supportive care. Treatments that increase herbicide clearance have been proposed including urinary alkalinisation (which increases renal clearance by ‘ion-trapping’) and haemodialysis (Bradberry et al., 2004). The toxicokinetics of the chlorophenoxy herbicides must be known to determine or interpret the effect of such interventions.

Animal studies of acute chlorophenoxy exposures demonstrate non-linear kinetics with high exposures due to dose-dependent changes in distribution and clearance for all herbicides within this group (Arnold and Beasley, 1989). MCPA is subject to dose-dependent saturation of protein binding *in vitro* (Roberts and Buckley, 2007a). While there is a prolonged apparent elimination half-life (*t*_1/2_) in animals with larger exposures it is unclear if this reflects decreased clearance or increased volume of distribution and whether the total and free concentrations are moving in tandem (Arnold and Beasley, 1989; Roberts and Buckley, 2007a; Roberts et al., 2005). It is necessary to better understand the dose-dependent kinetics in order to interpret changes after treatments that aim to increase clearance.

**Fig. 1:**
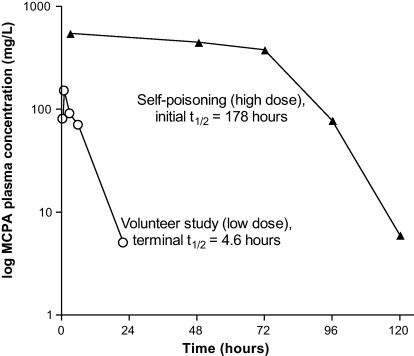
Semi-logarithmic plasma MCPA concentration–time using data from the literature: a case report of acute self-poisoning (Schmoldt et al., 1997) and a low-dose volunteer study (Kolmodin-Hedman et al., 1983).

Only two publications have described the kinetics of MCPA in humans, one was a single case of intentional self-poisoning (Schmoldt et al., 1997) and the other was a low-dose volunteer study (Kolmodin-Hedman et al., 1983). Comparison of the apparent elimination *t*_1/2_ from these reports may indicate that MCPA exhibits dose-dependent elimination (Fig. 1). The authors of this case report attributed the decrease in apparent half-life to treatment with alkaline diuresis (Schmoldt et al., 1997). However, a change in clearance was not directly quantified and dose-dependent changes in kinetics may explain the profile observed.

Details on the kinetics of MCPA are, therefore, of interest to guide research into the clinical management of acute poisoning. In particular, if the elimination of MCPA is confirmed to be prolonged in acute poisoning this will support research into treatments that enhance elimination. If the unbound concentrations are high this would indicate that haemodialysis might be effective. Here, we describe the plasma kinetics of MCPA in patients with acute intentional self-poisoning.

## 2 Materials and methods

### 2.1 Clinical

This is an observational study. Patients were identified by on-site study doctors on presentation to Anuradhapura or Polonnaruwa Hospitals with a history of acute poisoning. These hospitals provide 24-h medical and nursing care to patients. Patients were regularly reviewed and clinical details were recorded prospectively by on-site study doctors until discharge or death. All patients received supportive care which included supplemental oxygen, intravenous fluids, ventilatory and haemodynamic support as required. Antibiotics (usually penicillin and metronidazole) were given when aspiration pneumonitis was suspected clinically. There were no treatments involving urinary alkalinisation or haemodialysis due to resource limitations in the region.

Written informed consent was obtained in all patients who participated in this study. An admission blood sample was provided by patients followed by serial samples at 1, 4, 12, and 24 h, then once daily until discharge or death, as allowed by clinical factors. Blood was collected into an EDTA tube which was promptly centrifuged and the plasma was removed and frozen at −23 °C until the time of analysis.

Ethics approval for this observational study was obtained from Sri Lanka (the Universities of Colombo, Peradeniya and Sri Lankan Medical Association) and the grantholder's universities (Oxfordshire Clinical Research Ethics Committee (UK) and Australian National University).

### 2.2 Laboratory

The total and free (unbound) concentrations of MCPA were quantified in the samples collected above in addition to admission samples collected for a previous study (Roberts et al., 2005). The total MCPA concentration was measured in the above-mentioned plasma samples. 300 μL of plasma was then ultrafiltered using Millipore Centrifree Micropartition Device^®^ (Millipore, Bedford, MA, USA) yielding approximately 100 μg of plasma ultrafiltrate. The concentration of MCPA in the ultrafiltrate is the free (unbound) concentration.

The concentrations of MCPA were determined by Queensland Health Scientific Services (Australia) using a method derived from that of the United States Environmental Protection Agency (EPA, 1980). 100 μg of plasma or ultrafiltrate was hydrolysed in diluted sodium hydroxide and then buffered with acetic acid. The concentration of MCPA was determined by HPLC–MS/MS using an AB/Sciex API4000Q mass spectrometer in the negative ion mode equipped with an electrospray (TurboV) interface. This was coupled to a Shimadzu Prominence HPLC system (Shimadzu Corp., Kyoto, Japan) and a 50 mm × 2 mm C6-phenyl column (Phenomenex, Torrance, CA). The limit of reporting for the LCMSMS method was 1 μg/L for MCPA and the method was linear from at least 1–300 μg/L.

Method recovery was confirmed using MPCA concentrations of 2.5 mg/L to around 300 mg/L with an average recovery of 105% and a standard deviation 0.25. Therefore, the limit of detection (LOD; 3× standard deviation) is 0.75 mg/kg and the limit of reporting (LOR; using 6× LOD) is 4.5 mg/kg. The resulting concentrations (mg/kg plasma) were multiplied by 1.0205 which is the specific gravity of plasma at 37 °C (Trudnowski and Rico, 1974) to allow reporting with the unit mg/L.

To validate the Centrifree^®^ ultrafiltration device, plasma from a patient with MCPA poisoning was ultrafiltered, analysed for MCPA, re-ultrafiltered and then re-analysed for MCPA. There was no change in the concentration of MCPA between the 2 ultrafiltrates so MCPA does not appear to be adsorbed to the ultrafiltration device. Further, control plasma which did not contain MCPA was ultrafiltered and the filtrate was analysed for protein. It was noted that <0.2% of protein remained, suggesting that the Centrifree^®^ ultrafiltration device was efficient for removing protein from plasma samples.

The albumin concentration was determined in all admission samples and the concentration of albumin and creatinine was determined in all serial samples. These analyses were conducted by Queensland Health Forensic and Scientific Services at Princess Alexandra Hospital, Brisbane, Australia. This service is accredited by the National Association of Testing Authorities, Australia and certified to International Standards (ISO 9001).

## 3 Calculations

The MCPA concentration–time profile in patients providing the most serial samples was constructed using the total and free MCPA concentrations. A plot of the free versus total MCPA concentration was then constructed using data from all admission and serial plasma samples to determine whether protein binding was saturable and the approximate concentration at which this occurred.

The bound MCPA concentration was calculated as the difference between the free and total concentration at each time point. A Scatchard plot was constructed using the bound and free MCPA concentrations to estimate the number of apparent protein binding sites. Here, following visual inspection, a one-phase (linear) relationship suggests one-site binding, a two-phase relationship suggests two-site binding, and so on (Kermode, 1989; Molinoff et al., 1981; Motulsky and Christopoulos, 2005). In the case of two-site binding the relationship between free and bound concentrations is quantified by nonlinear regression using a two-site binding hyperbola model as follows:

$$\text{Bound concentration}={\text{Bmax}}_{1}\cdot \frac{\text{Cu}}{\text{K}{\text{d}}_{1}+\text{Cu}}+{\text{Bmax}}_{2}\cdot \frac{\text{Cu}}{\text{K}{\text{d}}_{2}+\text{Cu}}$$

Here, Cu is the free (unbound) plasma concentration of MCPA and Kd_*i*_ and Bmax_*i*_ are the affinity constant and maximum density (concentration of saturation) of binding at the *i*th site (Molinoff et al., 1981; Motulsky and Christopoulos, 2005). This analysis was initially conducted using the combined population data. To account for possible inter-individual variability in protein binding, the analysis was also conducted by global fitting. Global fitting is a computational regression method which incorporates best-fit data for individuals when determining the best-fit data for the group as a whole. Finally, because chlorophenoxy compounds largely bind to albumin (Braunlich et al., 1989; Rosso et al., 1998), this regression was also conducted relative to the concentration of albumin (g/L) to determine the extent to which this influenced the fit. When determining the protein binding properties of MCPA it was assumed that binding was at equilibrium at the time of measurement.

To determine whether saturation of protein binding influences clearance in humans the plasma apparent elimination half-life was determined prior to and following the concentration where saturation was calculated to occur. Data from patients providing two or more samples above or below the concentration of saturation were included and the best-fit apparent elimination half-life and 95% confidence interval was determined for the cohort. This was performed by non-linear regression with global fitting of the rate constant in a monoexponential decay model (*C*_*t*_ = *C*_i_ × exp(−*k* · *t*)). Here, *C*_i_ is the initial concentration and *C*_*t*_ is the concentration after time *t* when elimination occurs with a rate constant of *k*. In this analysis, *C*_*t*_ and *C*_i_ were allowed to vary between individuals to account for differences in exposure. Patients in whom the concentrations were not greater than the LOR in at least two samples were excluded from the kinetic analysis.

All regressions were conducted using GraphPad Prism version 4.03 for Windows, GraphPad Software, San Diego CA USA, www.graphpad.com.

## 4 Results

Serial samples were obtained in 33 patients and in 25 of these the concentrations were greater than the limit of reporting (5 mg/L) allowing inclusion in the analyses. All patients presented following acute intentional self poisoning and there was only one death. In the case of the survivors, regardless of the initial MCPA concentration, all survivors demonstrated signs of mild poisoning (predominantly nausea, vomiting and/or mild abdominal pain) and were discharged from hospital within 24–48 h (Table 1). The clinical sequelae of the patient who died have been reported previously (patient 7 in Table 2 (Roberts et al., 2005)). Briefly, this was a 45-year-old man with an altered level of consciousness who developed progressive tachycardia, tachypnoea, fever, haematuria and died 10 h post-admission to hospital. His treatment included intravenous fluids, endotracheal intubation and a single dose of sodium bicarbonate 25 mmol.

For all patients except three, the time of the maximum plasma concentration (Tmax) was noted on admission (Table 1). In the others the Tmax was at 3.7 h for two patients and 7 h post-ingestion in the third patient. This suggests that the absorption phase can be prolonged.

**Fig. 2:**
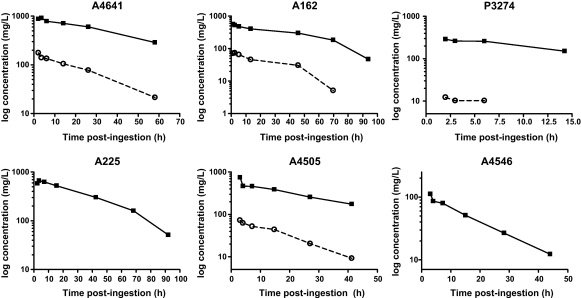
Semi-logarithmic plasma MCPA concentration–time curves for six patients with acute poisoning.

The concentration–time profiles for 6 patients with the highest number of samples are shown in Fig. 2. The initial rapid decrease in MCPA concentration in A4505 and A4546 possibly represents a distribution phase. An inflection in the semi-logarithmic concentration–time profile is observed in A162 and A225 producing a biphasic convex (downward-concave) curve (similar to that noted in rat administered high doses (Roberts and Buckley, 2007a)). A biphasic convex elimination curve was not obvious in the other patients, which may reflect the infrequent and short duration of sampling. In general, the free concentration mirrored the total concentration suggesting rapid equilibration between free and bound MCPA. Both curves are approximately log-linear which may suggest first-order elimination in this concentration range, however due to the limited frequency of sampling, zero order elimination cannot be excluded.

**Fig. 3:**
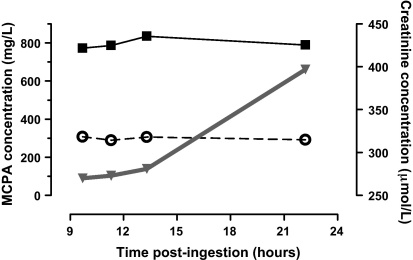
The MCPA and creatinine plasma concentration–time profile of a 45-year-old man who died 60 h post-ingestion.

The plasma concentration–time profile for the patient who died is shown in Fig. 3. It differed substantially to that of other patients shown in Fig. 2. During the 22 h post-ingestion the total and free MCPA concentrations did not decrease and the free concentration was the highest observed in this cohort. This plasma profile may occur when there is prolonged absorption, extensive distribution and/or impaired clearance. The admission albumin concentration in this patient was lower than that of the population (24 g/L compared with median 40 g/L, interquartile range (IQR) 36–42 g/L; *n* = 48) which would increase the free MCPA concentration and its distribution from the central compartment. Further, the plasma creatinine concentration in this patient was higher than others at admission (270 μmol/L compared with 95 μmol/L, IQR 83–116 μmol/L; *n* = 43) and increased until the time of death (Fig. 3). Such renal dysfunction would impair MCPA clearance (in contrast, the creatinine concentration in other patients fluctuated slightly or decreased during admission, data not shown).

**Fig. 4:**
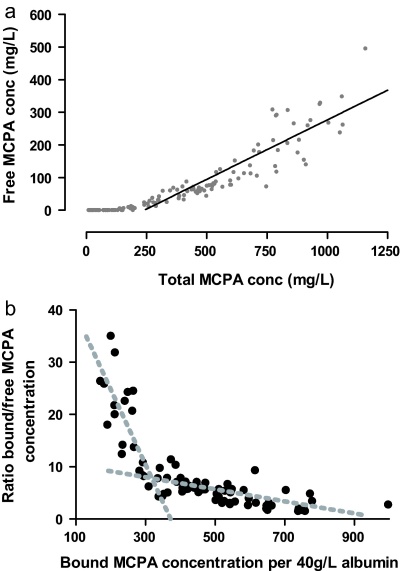
(a) Relationship between the total and free concentration of MCPA demonstrating saturable protein binding. (b) Scatchard plot showing a biphasic relationship which suggests binding of MCPA to two sites. Grey broken lines are freely drawn to indicate the two phases observed on visual inspection.

Protein binding characteristics were determined in 128 samples after excluding samples where the free concentration was less than the level of reporting. The free/total MCPA ratio increased when the total concentration exceeded 239 mg/L (95% confidence interval 198–274 mg/L) which is consistent with saturation of protein binding (Fig. 4a).

The Scatchard plot was approximately biphasic (in particular when the bound concentration was adjusted for the concentration of albumin), suggesting protein binding to two sites of differing affinity (Fig. 4b).

**Fig. 5:**
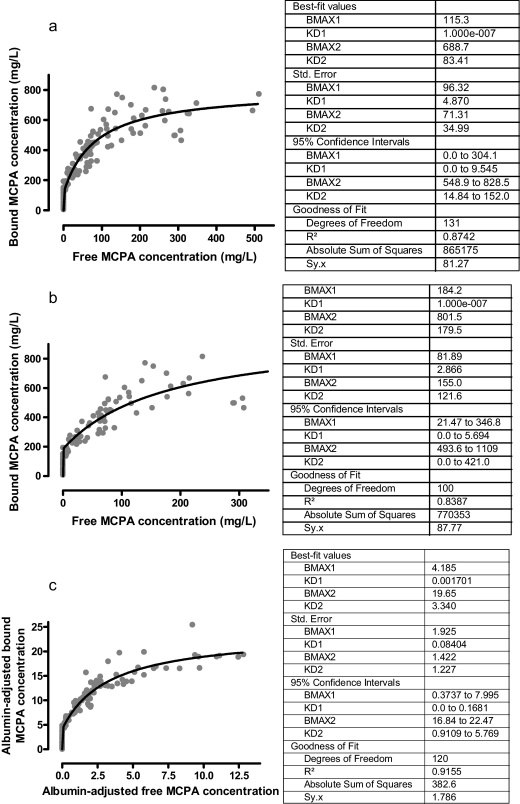
(a) Two-site binding hyperbolic relationship of free and bound MCPA in the aggregate population data. (b) Two-site binding hyperbolic relationship of free and bound MCPA in serial samples from individual patients using global fitting. (c) Two-site binding hyperbolic relationship of free and bound MCPA in the aggregate population data adjusted for albumin concentration.

Estimation of the characteristics of two-site protein binding using the aggregate population data suggested saturation of the high affinity binding site at a plasma MCPA concentration of 115 mg/L, but the 95% confidence intervals of the best-fit values were wide (Fig. 5a). Analysis by global fitting suggested saturation of the higher affinity binding site at a plasma MCPA concentration of 184 mg/L but the 95% confidence intervals were not markedly reduced (Fig. 5b). Analysis of the aggregate population data adjusted for the albumin concentration predicted saturation of protein binding at an MCPA plasma concentration of 4.2 mg/L per 1 g/L of albumin in plasma (167 mg/L using the median albumin concentration). Using this technique there was less scatter from the line of best-fit and the 95% confidence intervals were decreased for second binding site but not the first (Fig. 5c).

**Fig. 6:**
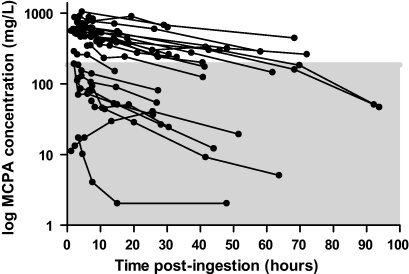
Concentration–time profiles for patients with sufficient plasma concentrations who survived (*n* = 24).

The concentration–time curves for all patients who survived are shown in Fig. 6. Based on the data presented in Fig. 5a–c, the high affinity protein binding site is saturated at a MCPA concentration less than 200 mg/L with a relatively wide 95% confidence interval. Using a tentative cut-off of 200 mg/L the apparent elimination half-life of the total concentration of MCPA during the initial phase (concentrations >200 mg/L) was 25.5 h (95% confidence interval 15.0–83.0 h; *n* = 16 patients). The terminal apparent elimination half-life at lower concentrations was shorter at 16.8 h (95% confidence interval 13.6–22.2 h; *n* = 10 patients). Data from the patient who died were not included in this analysis because a maximum plasma concentration was not apparent and the profile was atypical.

## 5 Discussion

This is the first description of the toxicokinetics of MCPA in a series of patients with intentional self-poisoning. MCPA displays two-site protein binding with saturation of the higher affinity binding site at a concentration less than 200 mg/L. This is within the concentration range typically observed in patients with acute poisoning, which can exceed 1000 mg/L (e.g. Fig. 6). When the concentration of MCPA exceeds the point of saturation, the free concentration increases rapidly and it is anticipated that MCPA will more readily distribute from the central compartment. The apparent elimination half-life at higher concentrations was 25.5 h which is slightly prolonged compared to the terminal phase of 16.8 h although the 95% confidence intervals of both estimations were wide. This long elimination half-life may contribute to the prolonged duration of poisoning observed in cases of self-poisoning, and slow elimination of MCPA may contribute to death. Therefore, more research is needed into the extent to which techniques for enhanced elimination, including urinary alkalinisation and haemodialysis, increase clearance and decrease the free concentration of MCPA.

The chlorophenoxy herbicides MCPA and 2,4-D display similar kinetic properties (Arnold and Beasley, 1989; Timchalk, 2004). Case reports of human self-poisoning have attributed a change in the apparent elimination half life of chlorophenoxy herbicides to treatment with urinary alkalinisation/diuresis (Flanagan et al., 1990; Friesen et al., 1990; Prescott et al., 1979; Schmoldt et al., 1997). On review of these cases it appears that the change in the apparent elimination half-life occurred when the concentration was approximately 150–300 mg/L, similar to Fig. 1. This is similar to the MCPA concentration where protein binding to the high affinity site appeared to saturate in our study (Fig. 5a–c) and also in rat studies (Roberts and Buckley, 2007a). Therefore, it is possible that the change in the apparent elimination half-life in Fig. 1 may have related in-part to the concentration-dependent change in toxicokinetics observed in our patients and in rat studies.

Regardless of the method employed, it is noted that the affinity of the first binding site for MCPA is extremely high and that it is saturated when the MCPA concentration is less than 200 mg/L (Fig. 5a–c). This confirms *ex vivo* studies that demonstrated the importance of albumin for MCPA–protein binding (Roberts and Buckley, 2007a). Given an albumin concentration of approximately 600 μM, if MCPA binds to albumin in a ratio of 1:1 then the binding sites are expected to be saturated at a concentration of 120 mg/L. We did not have sufficient high concentration samples to determine confidently if the second lower affinity site is potentially saturable with large exposures.

The p*K*_a_ of MCPA is 3.04, so the proportion of MCPA that is ionised (water soluble) decreases as the pH decreases (becomes acidic). This is reflected in the octanol solubility coefficient (Koc) which is a measure of lipophilicity. The Koc of MCPA is 1.0 at pH 6 and higher, but the Koc increases to 5.2 at pH 5 and then 45.6 at pH 4 (25 °C) (SciFinder, 2010). *In vitro* studies have confirmed enhanced cytotoxicity of chlorophenoxy compounds in an acidic medium, presumably due to an increase in cell penetration (Cabral et al., 2003). Therefore, urinary and potentially plasma alkalinisation in patients is likely to decrease the rate and extent of distribution due to ion-trapping.

Renal elimination is the most important route of clearance for MCPA (Fjeldstad and Wannag, 1977; Kolmodin-Hedman et al., 1983) but hepatic clearance may also contribute since glucuronidated metabolites are detected in the urine (Kolmodin-Hedman et al., 1983). MCPA is filtered, secreted and reabsorbed in the nephron and the extent of this varies between animal species, notably the rat and dog (Timchalk, 2004). Renal clearance also varies with hydration due to changes in urine flow (Proudfoot et al., 2004). With high MCPA exposures there is nonlinear renal clearance which may be due to saturation of active excretion (e.g., the renal organic anion transporting polypeptide) or direct nephrotoxicity (Koschier and Berndt, 1977; Pritchard et al., 1982; van Ravenzwaay et al., 2004). Penicillin may decrease MCPA clearance by competing for active secretion, as noted in rats (Braunlich et al., 1989); this antibiotic was not administered to the patient who died in this series. Nephrotoxicity in acute MCPA poisoning has been reported previously (Roberts et al., 2005) and may have contributed to the death of one of our patients given the progressive increase in creatinine (Fig. 3) and the very long elimination half-life.

Interpretation of elimination half-life is complex because it is a secondary kinetic parameter that depends on clearance (CL) and volume of distribution (Vd) which are related by half-life (*t*_1/2_), where *t*_1/2_ = 0.693 · Vd/CL. Further, the true elimination half-life can only be calculated once absorption and distribution are complete, which is difficult to determine following acute ingestion (hence the term ‘apparent’ elimination half-life) (Roberts and Buckley, 2007a). Biphasic convex concentration–time curves are observed in other poisonings which are susceptible to non-linear kinetics (Roberts and Buckley, 2007a). It is worthwhile determining physiological factors contributing to the biphasic convex plasma concentration–time profile because this may guide research into kinetic interventions for acute chlorophenoxy poisoning. Possible contributors include prolonged absorption, multi-compartmental concentration and pH-dependent distribution, saturable clearance or the influence of conjugated metabolites. This requires careful collection of both plasma and urine samples because the influence of dose-dependent toxicity or inter-individual variability in kinetics cannot be determined from plasma data alone.

Therefore, animal studies have demonstrated enhanced elimination of various chlorophenoxy compounds, including MCPA, with urinary alkalinisation (Braunlich et al., 1989; Hook et al., 1976). However, a systematic review failed to confirm the efficacy of this treatment in humans and it is not commonly used in countries where chlorophenoxy herbicide poisonings are frequent (Roberts and Buckley, 2007b). To confirm the effect of treatments that are proposed to increase the elimination of chlorophenoxy herbicides in humans, direct measurements of clearance are needed. In the case of urinary alkalinisation this requires measurement of the amount of the herbicide excreted in the urine and the extent to which this changes with pH. Of the limited number of cases where direct urinary measurements were conducted, urinary alkalinisation/diuresis appeared to be useful (Flanagan et al., 1990; Prescott et al., 1979). More research is required to further quantify the effects of urinary alkalinisation and to define the optimal treatment strategy. In patients with acute or chronic renal failure, other treatment strategies such as haemodialysis should be trialled.

## 6 Conclusions

MCPA exhibits dose-dependent protein binding within the range of concentrations seen in poisoning and possibly this leads to dose-dependence in other kinetic parameters. The full extent to which this occurs is not apparent from plasma concentration–time data only. Care must be taken when interpreting changes in kinetics (e.g. half-life) as a result of a treatment. More data on the kinetics of MCPA and other chlorophenoxy herbicides are needed, in particular mechanistic data determining if there is a significant increase in total clearance with haemodialysis or urinary alkalinisation.

## Conflict of interest statement

The authors D.M. Roberts, A.H. Dawson, L. Senarathna, F. Mohamed, and N.A. Buckley affiliated with South Asian Clinical Toxicology Research Collaboration (SACTRC) have been collaborated with the employees of Syngenta and Monsanto previously, which are manufacturers of herbicides. These collaborations have led to research publications in the peer reviewed literature and no personal payments were made to these authors.

## Figures and Tables

**Table 1:** Demographic and baseline data for 25 patients in whom multiple blood samples were obtained. Results shown as median and interquartile range.

Age (years)	25.5 (21.3–27.8)
Gender (M:F)	18:7
Time to present (h)	3.3 (2.5–6.0)
Duration of sampling (h)	40.8 (27.3–50.1)
Admission concentration (mg/L)	520 (186–654)
Outcomes (alive:death)	24:1
